# Blockchain-Based Forward Supply Chain and Waste Management for COVID-19 Medical Equipment and Supplies

**DOI:** 10.1109/ACCESS.2021.3066503

**Published:** 2021-03-17

**Authors:** Raja Wasim Ahmad, Khaled Salah, Raja Jayaraman, Ibrar Yaqoob, Mohammed Omar, Samer Ellahham

**Affiliations:** 1 Department of Electrical Engineering and Computer ScienceKhalifa University of Science and Technology 105955 Abu Dhabi 127788 United Arab Emirates; 2 Department of Industrial and Systems EngineeringKhalifa University of Science and Technology 105955 Abu Dhabi 127788 United Arab Emirates; 3 Heart and Vascular InstituteCleveland Clinic 2569 Abu Dhabi United Arab Emirates

**Keywords:** Blockchain, Ethereum, COVID-19, security analysis, forward supply chain, medical waste management

## Abstract

The year 2020 has witnessed unprecedented levels of demand for COVID-19 medical equipment and supplies. However, most of today’s systems, methods, and technologies leveraged for handling the forward supply chain of COVID-19 medical equipment and the waste that results from them after usage are inefficient. They fall short in providing traceability, reliability, operational transparency, security, and trust features. Also, they are centralized that can cause a single point of failure problem. In this paper, we propose a decentralized blockchain-based solution to automate forward supply chain processes for the COVID-19 medical equipment and enable information exchange among all the stakeholders involved in their waste management in a manner that is fully secure, transparent, traceable, and trustworthy. We integrate the Ethereum blockchain with decentralized storage of interplanetary file systems (IPFS) to securely fetch, store, and share the data related to the forward supply chain of COVID-19 medical equipment and their waste management. We develop algorithms to define interaction rules regarding COVID-19 waste handling and penalties to be imposed on the stakeholders in case of violations. We present system design along with its full implementation details. We evaluate the performance of the proposed solution using cost analysis to show its affordability. We present the security analysis to verify the reliability of the smart contracts, and discuss our solution from the generalization and applicability point of view. Furthermore, we outline the limitations of our solution in form of open challenges that can act as future research directions. We make our smart contracts code publicly available on GitHub.

## I. Introduction

The world has experienced unprecedented crises in 2020 due to the COVID-19 pandemic. For example, worldwide trade disruptions, slower economic growth, shortage of medical supplies, equipment, and hospitals, and an overwhelmed medical waste generation in hospitals. During the COVID-19 pandemic, a severe shortage of ventilators and personal safety equipment for medical staff has been reported in many hospitals in the USA [1]. A recent report reveals that the COVID-19 pandemic will cause the medical waste market to grow beyond 16.62 billion by 2023 at a compound annual growth rate (CAGR) of 3.8% [2]. The world health organization (WHO) estimated that 15% of medical waste is hazardous that can be infectious, radioactive, or toxic to the environment. The handling of such infectious or toxic medical waste produced by hospitals requires caretakers to comply with safety rules and regulations during the handling of such materials. The key objectives of such rules and laws are to ensure the safety of frontline health workers. For instance, inappropriate handling of the unprotected needles that are used to treat infectious diseases such as HIV or COVID-19 can cause the spreading of the disease [3]. Similarly, in the recent COVID-19 pandemic, improper disposal of the COVID-19 swab sticks or their use to test several patients can be dangerous since it can cause the spreading of the virus. Continuous monitoring of medical waste generated by COVID-19 infected patients and compliance of health provider’s actions with set rules using blockchain technology can assist to efficiently handle medical waste. The data and transactions stored on the blockchain are accessible to the stakeholders involved in the forward supply chain and waste management processes through distributed public or private ledgers. The decentralization feature of blockchain increases the trust among stakeholders as it eliminates the need to assess the trustworthiness of the participants [4]–[6].

**FIGURE 1.**
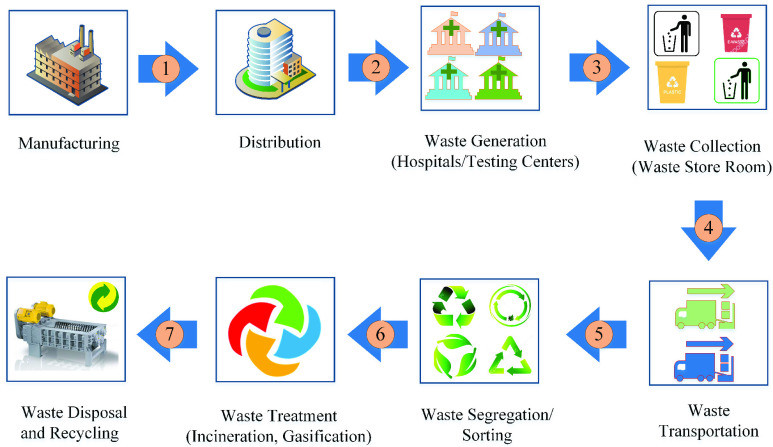
An overview of forward supply chain and waste management processes.

The forward supply chain involves the electronic ordering of medical equipment and supplies from manufacturing to consumption in the hospitals/COVID-19 testing centers. On the other hand, waste management of medical equipment and supplies refers to those processes that are involved in waste generation, collection, transportation, segregation, and disposal and recycling [7]. Traceability and operational transparency are the two main requirements of stakeholders involved in the modern forward supply chain and waste management practices of medical equipment [4]. The current COVID-19 medical equipment and waste are handled and managed by a vast network of manufacturers, distributors, retailers, shippers, storage facilities, suppliers, and waste treatment facilities who participate in the designing, production, shipment, sales, and disposal. The knowledge about what, where, and how the medical supplies and equipment are manufactured, originated, shipped, and used throughout their life cycle can significantly increase the trust among the involved stakeholders [4], [8]. On the other hand, the processes involved in waste management include collection, transportation, treatment, storage, disposal, and recycling of waste as highlighted in Fig. 1. Modern waste disposal and treatment practices such as incineration, bioremediation, and plasma gasification efficiently and systematically handle the massive amount of generated waste to improve public health, environment, and climate. The increased rate of urbanization, economic development, the world population growth, and the rise in the standard of living in developing counties are major causes of the amount, rate, and variety of generated waste [9]. The transactions related to waste management and establishment of data provenance about the medical equipment and supplies can be stored on the blockchain, which might otherwise be reliant on centralized or paper-based systems that are not trustworthy and prone to a single point of failure or manipulation [4].

Most of the current technologies leveraged for managing forward supply chain and waste disposal are mainly based on cloud computing and the Internet of things (IoT) to cope with domestic, electronic, and biodegradable waste problems. For instance, the studies conducted in [10] and [11] employ IoT technology to monitor and report the available storage capacity of the waste bins deployed within a community. After collecting waste bin data using IoT sensors, it is stored on a central control server that processes it to identify and enlist those bins whose remaining storage capacity is below a certain threshold. It triggers a notification to the appropriate shipper through a route optimization algorithm to ship the waste. The existing cloud and the IoT-based systems used to manage the forward supply chain of medical equipment and their associated waste are incompetent in providing operational transparency, audit, verifiability, traceability, and identifying fraudulent documentation. Also, they are centralized that makes them vulnerable to a single point of failure problem. In this paper, we propose a blockchain-based solution that can manage the COVID-19 related medical equipment along with their supplies and waste disposal after usage. We implement safety rules to minimize the spreading of COVID-19. Through auditing service, our proposed solution issues penalties to the users for any non-compliance with COVID-19 related rules. Our study does not focus on reverse supply chain processes [7] as the COVID-19 related medical equipment and supplies (e.g., PPE, swab sticks and swab sample test tubes) are not recyclable to avoid the risk of spreading of COVID-19. The main contributions of this paper are as follows:

• We showcase an Ethereum blockchain-based approach to trace and track the COVID-19 medical equipment and supplies and their waste after usage in a decentralized, secure, transparent, auditable, and trustful manner.
• We develop smart contracts along with algorithms to automate and implement the services for the forward supply chain management of COVID-19 related medical equipment and supplies, their waste shipment, and assurance of compliance with safety measures to minimize the spread of COVID-19. We employ decentralized storage of interplanetary file systems (IPFS) to overcome the blockchain storage limitations.
• We test and validate the proposed approach against many functionalities/services such as medical equipment order handling, ownership transformation, inventory management, waste shipment management, real-time waste monitoring, issuance of penalties to stakeholders to assure safety compliant operations, to assess their correctness, practicality, and feasibility. We report the logs on executing the transactions on the blockchain for each functionality.
• We present transaction execution and computational cost and security analysis of each function in the implemented smart contracts to demonstrate the affordability and reliability of the proposed solution. We outline several important challenges and limitations acting as future directions for new researchers.
• We propose a generic prototype solution that can be implemented on both public and private blockchains with minimal modifications based on the requirements and preferences of existing healthcare industries. Our implementation code can be publicly accessed via GitHub repository.^1^

^1^https://github.com/AhmadKhalifaUniversity/Code/blob/main/Registration

The remainder of the paper is organized as follows. Section II discusses the related work. Section III presents the proposed system along with sequence diagrams to show the interactions of stakeholders with the system. Section IV presents the implementation details. Section V tests and validates the system for a set of scenarios. Section VI presents a discussion on security and cost analysis and related research challenges. Section VII concludes the paper.

## II. Related Work

This section briefly discusses the recent developments and proposals related to the forward supply chain of medical equipment and management of domestic and electronic waste.

The forward supply chain processes of medical products (e.g., equipment, devices, and medicines) aim to deliver medical products timely and reliably to end users from the source. The technology-driven approaches including RFID tags, bar codes, near field communication (NFC), and serialization can assist to track the medical products throughout their forward supply chain phase in the healthcare industry [12]. Blockchain-based solutions employed in the healthcare industry assist securely trace and track the medicines from manufacturer to distributors to patients. In [13], authors exploited the Gcoin blockchain to design a system that can trace the drugs throughout their supply chain process. It has digitized the operations involved in business processes among manufacturers, wholesalers, pharmacies, and drug consumers. The immutable logs of the transactions stored on blockchain enable authorities to identify the counterfeit medicines supplied to the hospitals. In [14], researchers have proposed a framework that can provide safety to drugs, and assures that the purchased medicines are manufactured by an approved manufacturer. Establishing provenance data about the medical equipment, similar to medical drugs, is the main concern of the authorities as safety and quality of medical equipment can affect human lives. In [15], [16], authors identified and presented the participants involved in blockchain-based systems to trace a medical device.

Traditional waste management systems do not offer incentives to people to produce less amount of waste than what they are producing. In [17], a flexible pricing and environmentally friendly waste management system named Smart waste management system (SWMS) was proposed. It introduces a weight-based price estimation model to charge the service cost to the waste producers. The SWMS employs IoT technology to monitor, measure, and collect waste-related data about waste bins located in different geographical regions. The central servers are designated to provide services to those stakeholders (e.g., transportation companies) that need fast read/write operations. The blockchain was used to assist the users to make micropayments in a transparent and trusted way with the least financial transaction cost overhead. The drawback of the system is its incompetence to handle the man-in-middle and DoS attacks in centralized-based services.

In [18], an Ethereum-based system called Electronic waste management (EWM) was proposed that assures compliance with waste disposal guidelines for electrical and electronic equipment (EEE). The main stakeholders considered by the proposed system for automating the EEE waste management services include producer, consumer, and retailer of electronic components. The smart contracts calculate, record, report, and provide incentives to the consumer for returning the EEE waste to the retailer. The retailer-centered smart contracts verify that the waste is received for all sold EEE. The retailer ships the collected waste to the EEE producer and assures that a portion of the EEE original cost is paid to the consumer. The smart contracts calculate and impose the penalties to the producer if waste is not collected from retailers within the predefined period.

In [19], the authors proposed a 5G-enabled blockchain-based system that assists in tracking electronic products throughout their lifetime. The system employs an Ethereum blockchain platform to implement and present a working prototype of the system comprising of the manufacturer, supplier, retailer, customer, and E-waste facility unit. Smart contracts of the E-waste facility unit calculate and give incentives to customers for channelizing their waste to dispose of it at the E-waste facility. It requires all stakeholders to deposit escrow (equivalent to the tax amount of the product) in a smart contract wallet to force them to comply with the set rules. The deposited amount along with incentives is returned to the stakeholders when waste reaches the facility after evaluating the stakeholder’s compliance with rules. The performance assessment of the system in terms of system gas consumption and throughput was not discussed in the study.

In [20], a working prototype of an Ethereum-based system is proposed that assists the users in monitoring waste of solid materials (e.g., computers and smartphones). Unlike [18], [20] can continuously monitor and track waste on its transportation through trucks to the waste storage factory. Key external stakeholders of the architectural design of the proposed system include a collection manager, storage manager, transaction manager, and treatment manager. The collection manager generates and stores the QR code that refers to a package holding solid waste material on the blockchain. The transportation manager records the state, location, and route information about the vehicle transporting solid waste. The proposed system has used off-chain storage systems to temporarily store encrypted data related to solid waste. The system can be made more proficient by employing multiple blockchain platforms to keep the balance between security and throughput.

In [21], a prototype of an Ethereum-based system that can track the waste across the border in a secure, tamper-proof, and privacy-preserving way was presented. The sensitive and non-disclosable information are stored locally to preserve the privacy of data. For audit-critical data such as e-waste type, assets, handler, and log of executed processes during e-waste processing, treatment, and handling, it digitally signs the data and transactions to further protect it. Through smart contracts, authorities can audit and verify the reasoning of delay in reaching transportation between exchanged locations, the volume of transportation, and the validity of waste handling methods. This study can be further enhanced by introducing incentives and penalties into it to force the participating entities to observe the waste management rules.

The lack of operational transparency can cause frauds that occur during the waste collection stage. The study conducted in [22] proposes a framework for the traceability of personal protective equipment (PPE) for medical staff combating with COVID-19. In [23], the authors presented a system that uses an image analysis-based technique to identify the frauds related to waste collection. The proposed study calculates the similarity index between the two images that are captured at the garbage collection point and waste bin tray, estimate the weight of garbage, location, and time differences of two captured images, to identify waste-related frauds. After calculating such data, it incentivizes the users. It employed blockchain technology to securely transfer the cryptocurrency to the users as an incentive for collecting the waste. Another study conducted in [24] has proposed a blockchain-based system to keep track of all remanufacturing/refurbishing processes to smartphones. However, the authors have not discussed a solution to verify the frauds related to remanufacturing/refurbishing processes.

In summary, none of the aforementioned studies has highlighted the need for improved transparency, efficiency, and trust in the forward supply chain and waste management of COVID-19 medical equipment. In contrast, this research study proposes a blockchain-based solution that can assist the stakeholders to trace and track the medical equipment such as swab sticks and swab sample test tubes to verify their disposal in a secure, transparent, trustful, decentralized, and auditable way.

## III. Proposed Blockchain-Based Solution

**FIGURE 2.**
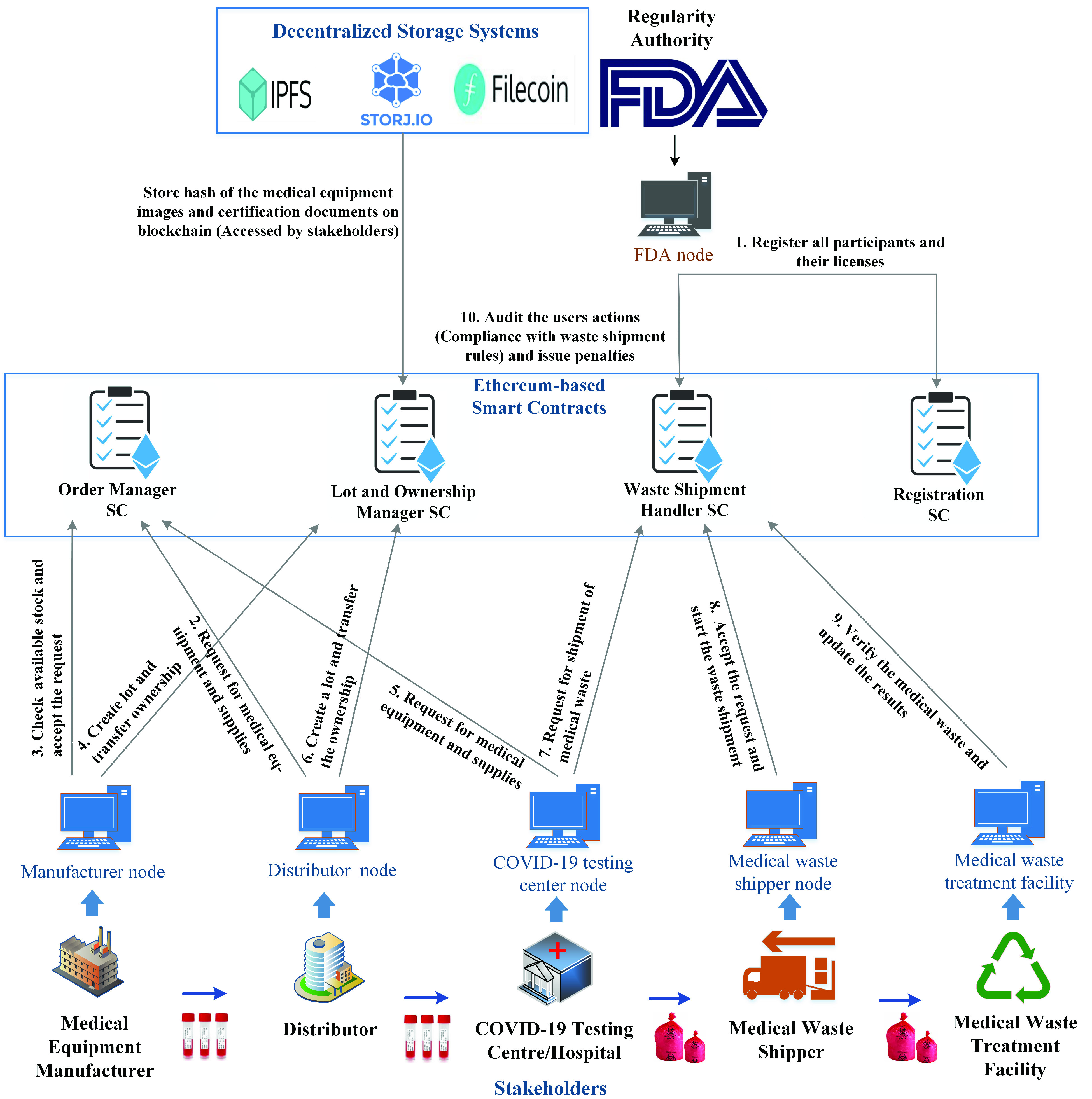
An overview of the proposed blockchain-based system for the forward supply chain and waste management of COVID-19 medical equipment.

In this section, we present a high-level design of the proposed blockchain-based solution to manage COVID-19 medical equipment supplies and their waste after usage. Fig. 2 highlights the main stakeholders, system components, and actions of the stakeholders involved in the management of medical equipment and their waste part. Further discussion is provided in the following section.

### A. System Participants and Components

Herein, we briefly explain the responsibilities of each participant involved in the forward supply chain of COVID-19 medical equipment and medical waste generation, handling, monitoring, and shipment. It also discusses the key components of the system that facilitates the secure sharing of data among participating stakeholders.

#### 1) COVID-19 Medical Equipment Manufacturer

The role of a medical manufacturer is to store details about the newly manufactured medical equipment and supplies on the blockchain. The most important data about the manufactured medical equipment and supplies include product name, type, cost, credentials of the manufacturer, manufacturing date, design, composition, and certifying agency. A manufacturer normally researches the current need and requirements of the market to develop medical equipment. It surveys the market to find the trusted raw material supplier and places an order for the supply of raw material to manufacture medical equipment and equipment. It assures that all necessary documents such as specifications and certification records regarding all manufactured medical equipment are available. Also, the manufacturer should be genuine and all the details about the valid license should be publicly made available.

#### 2) Distributor

The responsibility of the distributor in the forward supply chain of medical equipment and supplies used in COVID-19 screening centers is to revisit the market demand to purchase equipment from the registered and highly reputed manufacturers. Thus, it acts as an intermediary between medical equipment consumers such as COVID-19 screening centers or hospitals and medical equipment manufacturers. A distributor aims that the order placed by the hospitals for the medical equipment and supplies should be immediately fulfilled. It is also responsible to create and prepare the lot of medical equipment and ship them to consignees. It needs to be ensured that the distributor should have a valid license to purchase and sell medical equipment and supplies.

#### 3) COVID-19 Testing Center/Hospital

COVID-19 testing centers/Hospitals are one of the consumers of medical equipment and supplies (COVID-19 swab sticks). Medical equipment and supplies are normally purchased from the distributor or they are directly shipped from the manufacture. COVID-19 testing centers/Hospitals generate medical waste on testing the public for COVID-19 using medical equipment and supplies such as nasal swab sticks. The hospital staff temporarily stores medical waste in a safe storage room. The waste handlers and nursing staff makes sure that the waste is managed in a safe way to comply with standard operating procedures to deal with the COVID-19 pandemic. Later on, this medical waste is transported to the waste treatment facility for processing and disposal.

#### 4) Medical Waste Shipper

The medical waste generated in hospitals/COVID-19 testing centers is shipped to the waste treatment facility via third-party shipping services for treatment and disposal. To comply with the COVID-19 standard operating procedures, medical staff, and medical waste shippers having COVID-19 symptoms are not allowed to handle medical waste. However, those staff and shippers who have received COVID-19 vaccination can handle medical waste. Finally, it should be assured that the bags carrying COVID-19 medical waste are sealed properly, not mixed up with general waste, and secure enough against human interaction during its shipment.

#### 5) Medical Waste Treatment Facility

The main responsibility of the manager in a medical waste treatment facility is to ensure safe interaction with medical waste during its treatment and disposal stage. At the medical waste treatment facility, COVID-19 medical waste is handled carefully especially during the waste segregation and processing phase to comply with the standard operating procedures devised to interact with COVID-19 waste. The medical equipment including nasal swab sticks and swab tubes are disposed of at the medical waste treatment facility since they are not recyclable, thereby minimizing the chances of spreading COVID-19 disease.

#### 6) Regularity Authority

The responsibility of the regularity authority such as the Food and Drug Administration (FDA) is to register (on-chain) each stakeholder (license) along with their role in the forward supply chain of COVID-19 waste delivery. It also assures that stakeholders perform actions in compliance with defined rules and regulations. Moreover, it imposes penalties on the stakeholder when an action that is non-complying with the stated rules is noticed. For instance, it can suspend the license of a participant such as a shipper if the COVID-19 waste is not properly handled during its disposal or shipment.

#### 7) Distributed Storage

The participants of the medical waste management framework can store large-sized files on the distributed storage systems, such as IPFS, to overcome the scalability issues of blockchain [25]. In our proposed system, large-sized files related to COVID-19 medical equipment and supplies management include medical equipment certification and specification files. These files are stored on the IPFS and the hash of such files is stored on the blockchain ledger. The immutability feature of blockchain enables the users to verify the correctness of stored documents on the distributed storage systems.

#### 8) Smart Contracts

Smart contracts replace the intermediaries and assure the governance of rules as agreed upon by the participating entities [26]. In this paper, we implement four major smart contracts; namely, order management smart contract, the lot and ownership management smart contract, Waste shipment handler smart contract, and registration smart contract. All these smart contracts constitute a set of functions that are called when a certain event happens in the COVID-19 medical equipment supply chain.

### B. Sequence of Operations

This subsection explains sequence diagrams showing the function calls and blockchain-based events to automate the processes involved in the traceability of COVID-19 related medical equipment and their associated waste disposal.

**FIGURE 3.**
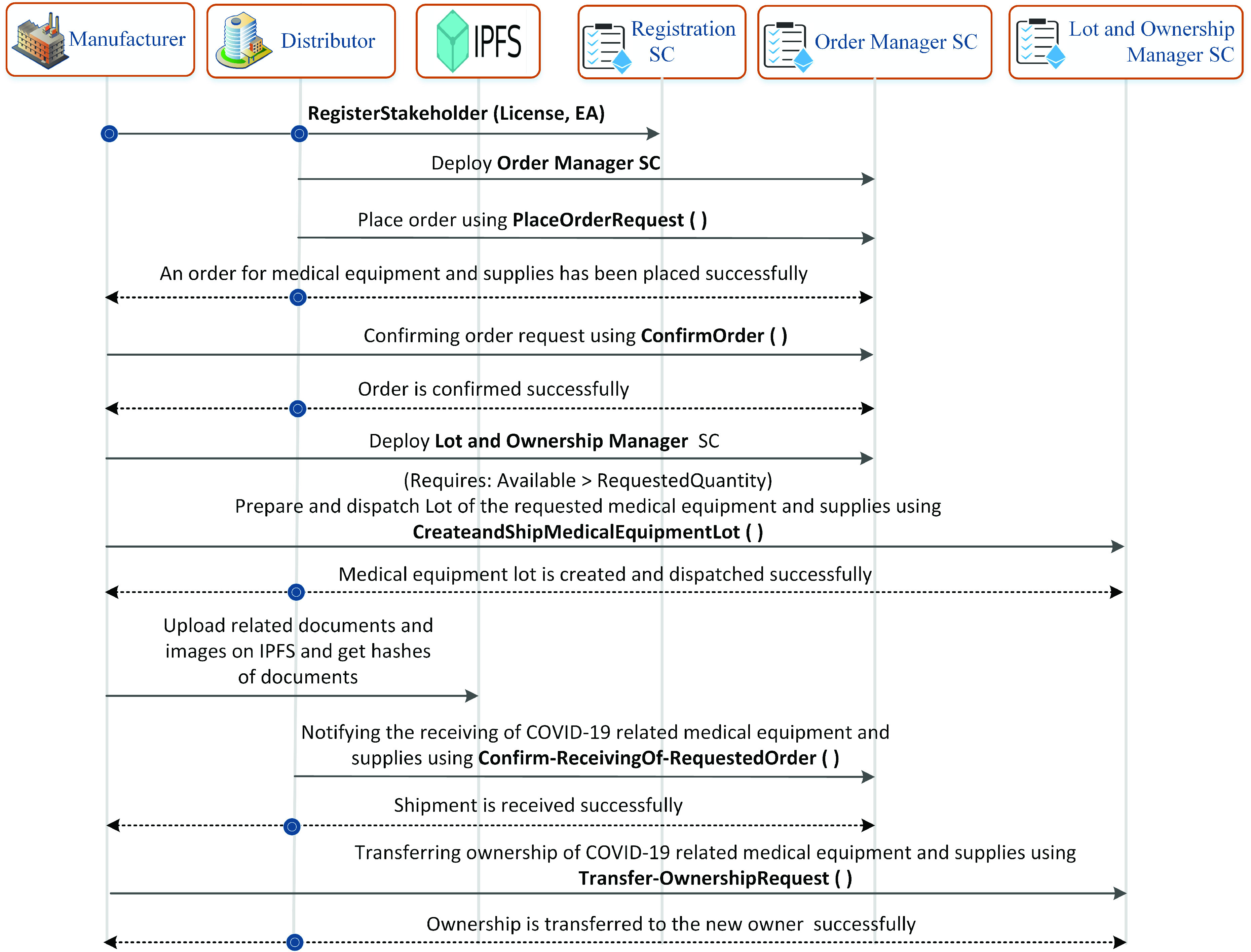
Sequence diagram showing the function calls and events between manufacturer and distributor.

The sequence diagram shown in Fig. 3 highlights the user’s interaction with the system. All the participants are registered on the blockchain using a registration smart contract which is stored on the blockchain. The successful registration gives a unique identifier to the stakeholder which needs to be used in future for referring to the user or the services which are associated with it. After successfully registering a user on the blockchain platform, the distributor calls a function to deploy the order manager smart contract. The order manager smart contract is responsible for assisting the caller to place, confirm, or reject an order related to COVID-19 medical equipment and supplies. After a successful deployment of the order manager smart contract, the distributor stakeholder calls *PalceOrderRequest* function that as a result successfully places an order for the manufacturer to arrange medical equipment and supplies such as COVID-19 swab sticks. As a response, the manufacturer calls *ConfirmOrder* function to accept/reject the medical equipment order request by the distributor after analyzing the available medical equipment and supplies stock. Manufacturer prepares a lot of medical equipment and supplies and stores the details such as lot ID, quantity, name, type, certification number, and quality of the equipment on the blockchain platform. It also uses the IPFS system to store large-sized medical equipment certification documents and images of the shipping packages. It stores the hash of these documents on the blockchain to assure the authenticity of medical equipment. On successfully receiving the medical equipment lot, the distributor notifies the manufacturer by calling *Confirm-ReceivingOf-RequestedOrder* function. Finally, the distributor is promoted as the new owner of the medical equipment lot by the manufacturer.

**FIGURE 4.**
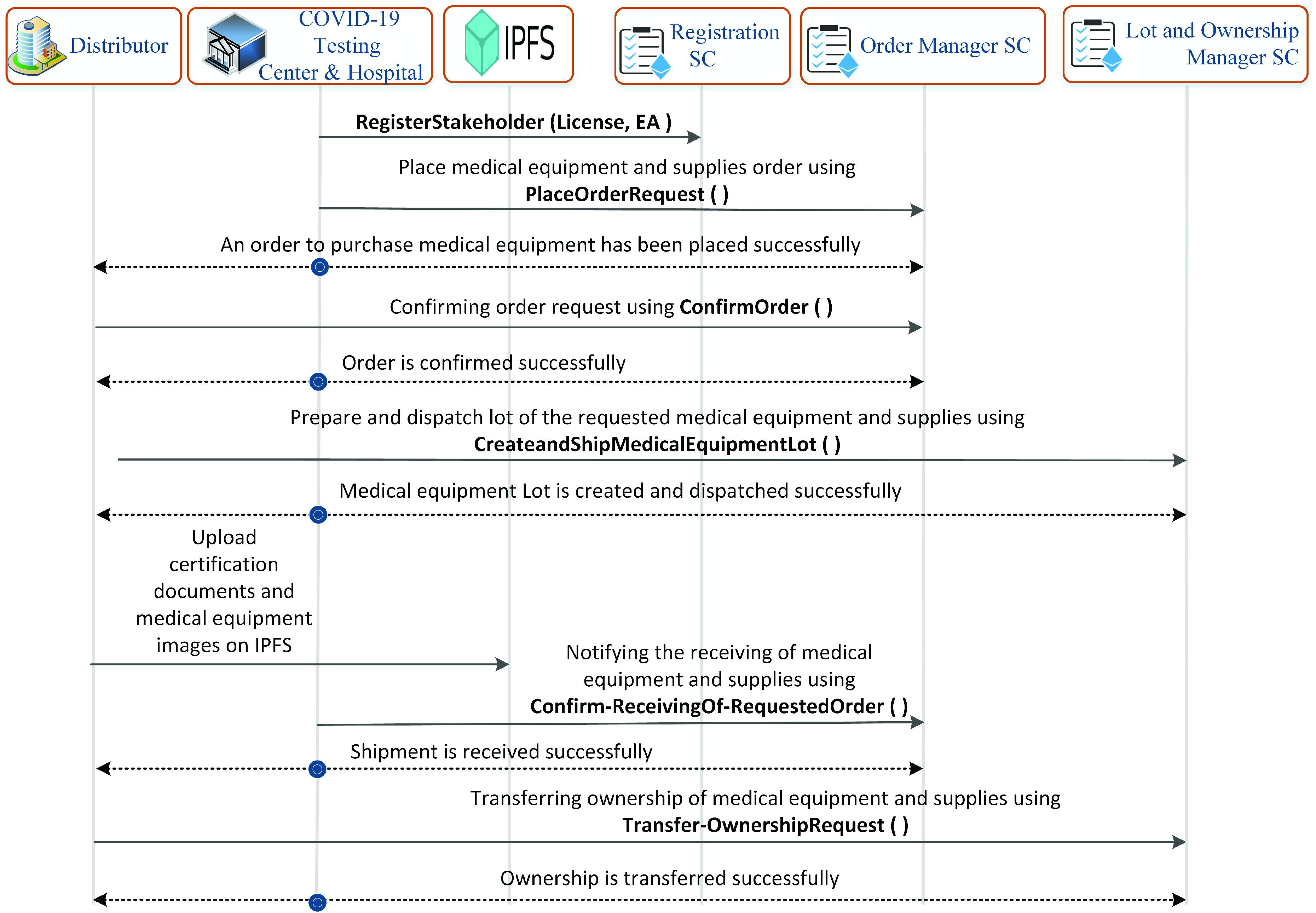
Sequence diagram showing the function calls and events between distributor and COVID-19 hospital & testing center.

**FIGURE 5.**
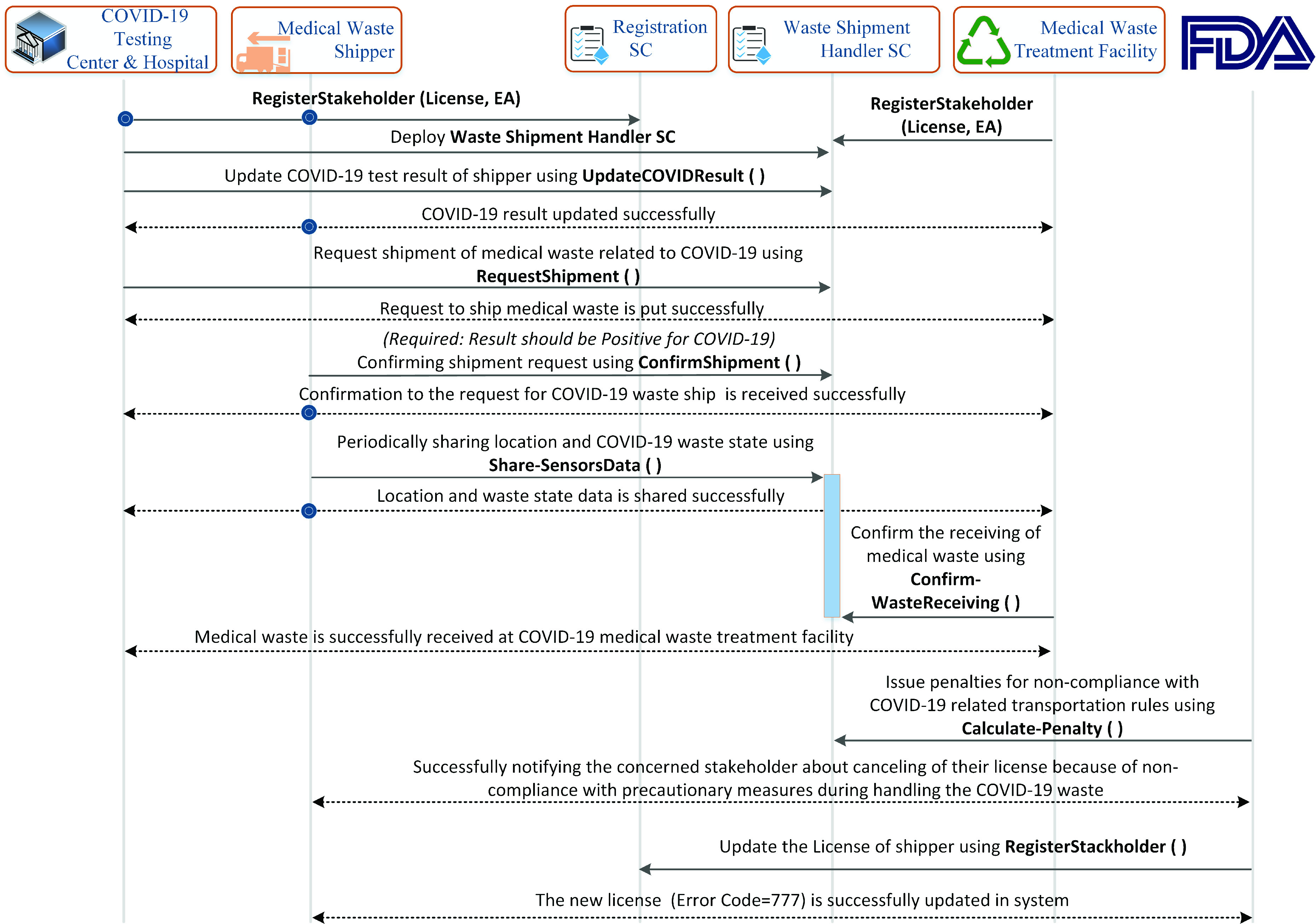
Sequence diagram showing the function calls and events among COVID-19 hospital & testing center, medical waste shipper, FDA, and medical waste treatment facility.

Fig. 4 captures the interaction between COVID-19 testing center/hospital and medical equipment distributor. Each entity in these systems is registered and represented by a unique Ethereum address. The call to *PlaceOrderRequest* function in order manager smart contract by the COVID-19 testing center places an order to the distributor to prepare and ship the medical equipment. In response, the distributor calls *ConfirmOrder* function to notify the COVID-19 testing center about the availability of requested medical equipment. An event is triggered by the smart contract to notify all related stakeholders about the response of the distributor. The distributor entity creates the lot and assigns new labels to the existing packages to ship it to the COVID-19 testing center. It also triggers *Transfer-OwnershipRequest* function to make COVID-19 testing center a new owner of the medical equipment lot. In contrast to Fig. 3 and Fig. 4, Fig. 5 captures the interaction between those stakeholders that control and manage COVID-19 equipment related to medical waste. The COVID-19 testing center calls *ShipmentRequest* function to place a request to the certified shippers to ship the medical waste to the medical waste treatment facility. The shipper, in response, confirms its availability by calling *ConfirmShipment* function. Also, the shipment management smart contract notifies all the related entities about the response of the shipper. To comply with precautionary measures taken to mitigate the spread of COVID-19, waste bags are required to not be opened during its shipment. The shipper continuously stores state and location data on the edge server. In response, it calls *Share-SensorsData* function to store the location and state of the medical bags on the blockchain. The state of the waste bag indicates whether the bag was opened during the shipment or not. The smart contract notifies the relevant entities such as the medical waste treatment facility and COVID-19 testing center about the updated data to track the location and state of the medical waste during its shipment. This data is used by the FDA to verify that the COVID-19 medical waste was handled properly during its transportation to the medical waste treatment facility to minimize the spread of COVID-19. It cancels the license of the shipper if the COVID-19 medical waste is not handled properly.

## IV. Implementation Details

This section presents the implementation details of the proposed blockchain-based approach. We use an Ethereum platform to implement smart contracts to govern the business activities of stakeholders. We use Remix IDE to develop, deploy, and test the smart contracts. The smart contracts developed to digitize the services of participating stakeholders include registration, lot and ownership management, order management, and shipment management.

Algorithm 1 presents a procedure to place an order request to the provider of COVID-19 related medical supplies and equipment. It requires the caller to provide input data such as the Ethereum address of the receiver of the medical supplies and equipment, medical supplies quantity required, the Ethereum address of each requested medical equipment, name, and type to successfully initiate the request and notify the participating stakeholders. It assures that only distributor or COVID-19 screening centers/hospitals can initiate a request for the medical supplies and equipment. It rejects any request for the medical supplies if initiated by any unregistered and unlicensed distributor/COVID-19 screening centers/hospitals. It records the order details such as type of medical equipment and supplies requested, requested quantity, OrderID, and Ethereum addresses of the receiver, equipment, and the orderer stakeholders. It employs the secure one-way hash function called Keccak256 that uses encrypted strings to generate a unique, collisions free, and irreversible identifier. Such a unique identifier to the medical equipment and supplier order can be used to track and trace medical equipment and supplies order during its forward supply chain management operations. The Keccak256 hash function is the native algorithm used in Ethereum classic and other Ethereum platforms. Moreover, it is fast, resource-efficient as it does not have specific hardware requirements, and exhibits a special property called avalanche effect that makes it highly secure. In our solution, *Abi.EncodedPacked* function is used to generate a padded byte string of the input data including caller EA, time, equipment EA, equipment type, and name. The Keccak256 hash function uses this byte string to create a unique reference for each medical equipment and supplies order. On successful execution, Algorithm 1 notifies all the participating stakeholders about the awaiting/pending request by emitting an event about registering the recent order placement request.

**Algorithm 1:**
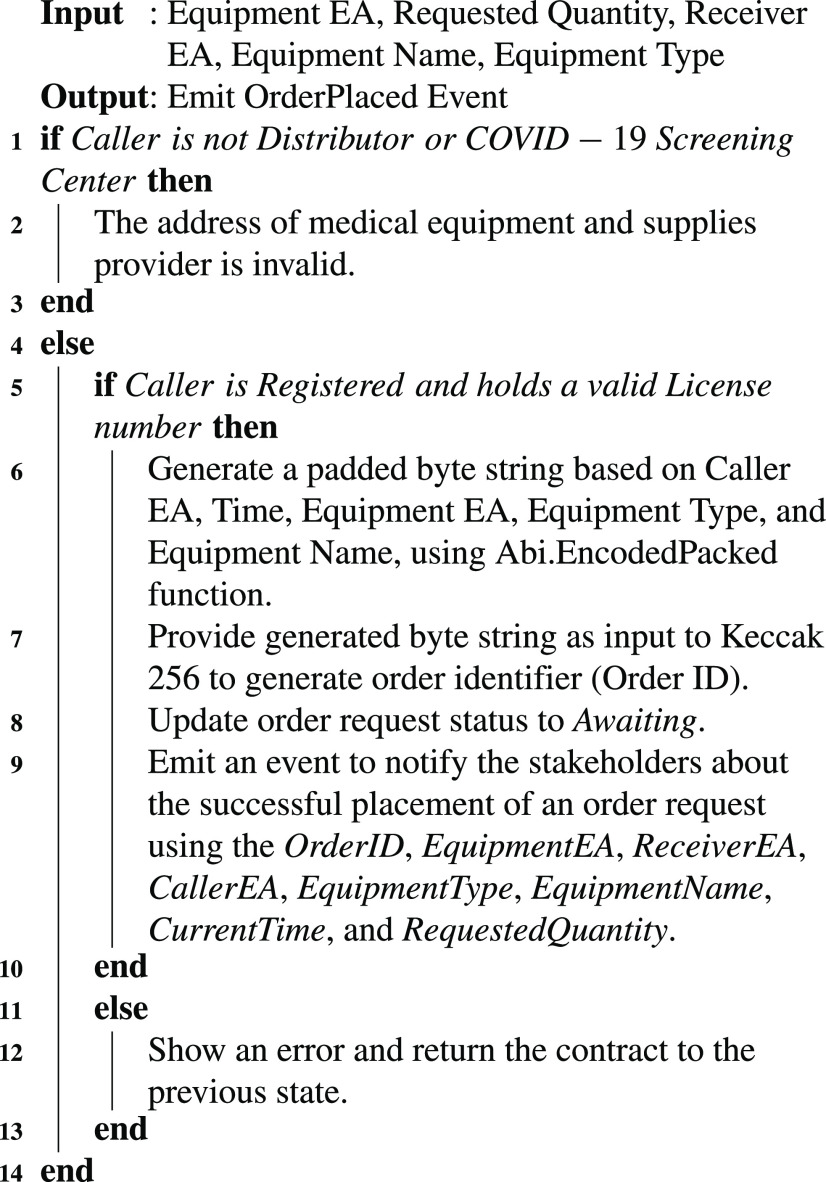
Placing an Order for Medical Equipment and Supplies

The pending medical supplies and equipment requests are accepted or rejected by the distributor/manufacturer. Algorithm 2 highlights the business flow along with functionalities performed in selling the medical supplies to the orderer. It assures that only registered distributors and manufacturers with a valid license number can sell the medical supplies to the orderer for minimizing the fake drugs supplying from the unlicensed manufacturer or distributor. The lock variable protects the system from reentrancy attacks. The initial value of the lock is set to a non-zero integer value referred to as Item. Before and after executing statements that manage the medical equipment and supplies stock, the lock variable is updated accordingly to assure that only one instance of a user can access such data at a time. After preparing the medical equipment and supplies lot to ship to the orderer, the remaining medical equipment and supplies stock is updated by subtracting the requested quantity of medical equipment and supplies from the total available medical supplies stock. The participating stakeholders are notified about the sold medical equipment quantity and recipient of the order by emitting an event to record Ethereum addresses of seller and receiver, sold quantity, and current wall clock time.

**Algorithm 2:**
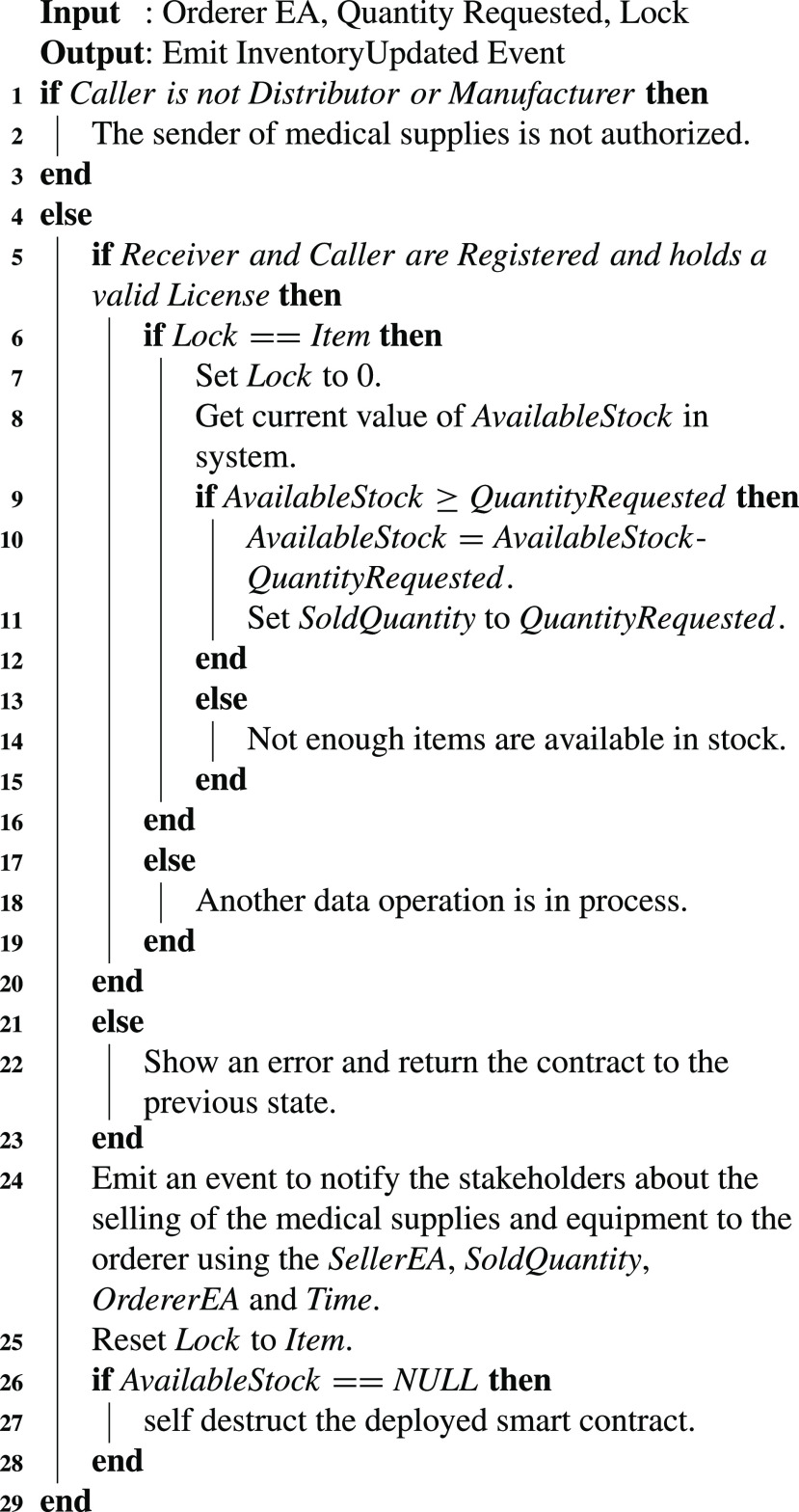
Updating the Medical Equipment and Supplies Inventory

Medical equipment is used for screening, swab checking, and providing treatment to the people suffering from COVID-19. The medical waste is generated once such medical supplies or equipment (e.g., COVID-19 swab sticks) are used in hospitals. This medical waste requires an immediate service to ship it to the medical waste treatment centers for disinfection and disposal. Algorithm 3 highlights the procedure along with system functions and logs to place a medical waste shipment request. This algorithm can only be triggered by the COVID-19 testing centers/hospitals as shown in step 1 of Algorithm 3. It assures that the potential shippers of the COVID-19 medical waste should be registered, they have a valid license and recently tested negative for COVID-19 to further minimize the chances of COVID-19 spreading. A shipment identifier is created using a hashing algorithm (SHA-3) along with a pending shipping status to track the shipment request. The secure hashing algorithm (SHA-3) has two versions; namely, Ethereum SHA-3 and the official SHA-3 standard. The former version is also known as Keccak256. In our proposed approach, each waste shipment order is referenced by a unique shipment identifier which is generated by the Keccak 256 hash function. Keccak 256 generated such an identifier by using an encoded byte string. Many parameters such as shipper EA, COVID-19 screening center EA, weight of waste, waste pickup time, and current time, are used as input to the *Abi.EncodedPacked* function to produce the encoded byte string. This unique identifier serves as a tool to trace and track the medical equipment and supplies waste to assure that it is handled in compliance with COVID-19 related rules to restrict the virus from spreading. The shipment order request to the waste shipment company includes a set of parameters including waste pickup time, waste pickup location, order status, shipper address, the weight of the shipment package, a hash of the medical waste images, sensors state, and unique identifier of the medical equipment waste. The data collected using sensors attached to the waste bags are referred as sensors state data in Algorithm 3. The sensors continuously monitor the state of COVID-19 waste bags and issue a transaction on the blockchain (i.e., violation count) on identifying any unauthorized attempt to open the COVID-19 waste bags. Similarly, the status order variable as highlighted in Algorithm 3 represents the most recent status of the waste shipment order, which can be pending, accepted, rejected, shipping, reached, and received, depending on the COVID-19 waste management stage. After the successful execution of Algorithm 3, the variable status order is set as pending. The COVID test result variable holds the most recent COVID-19 test result of the shipper. The statusFlag parameter is set as false in Algorithm 3. This variable is used by the smart contract functions to verify that the request for the waste shipment is placed successfully by the COVID-19 screening center. On successful execution, it notifies the related stakeholders about the details related to this transaction by emitting an event.

**Algorithm 3:**
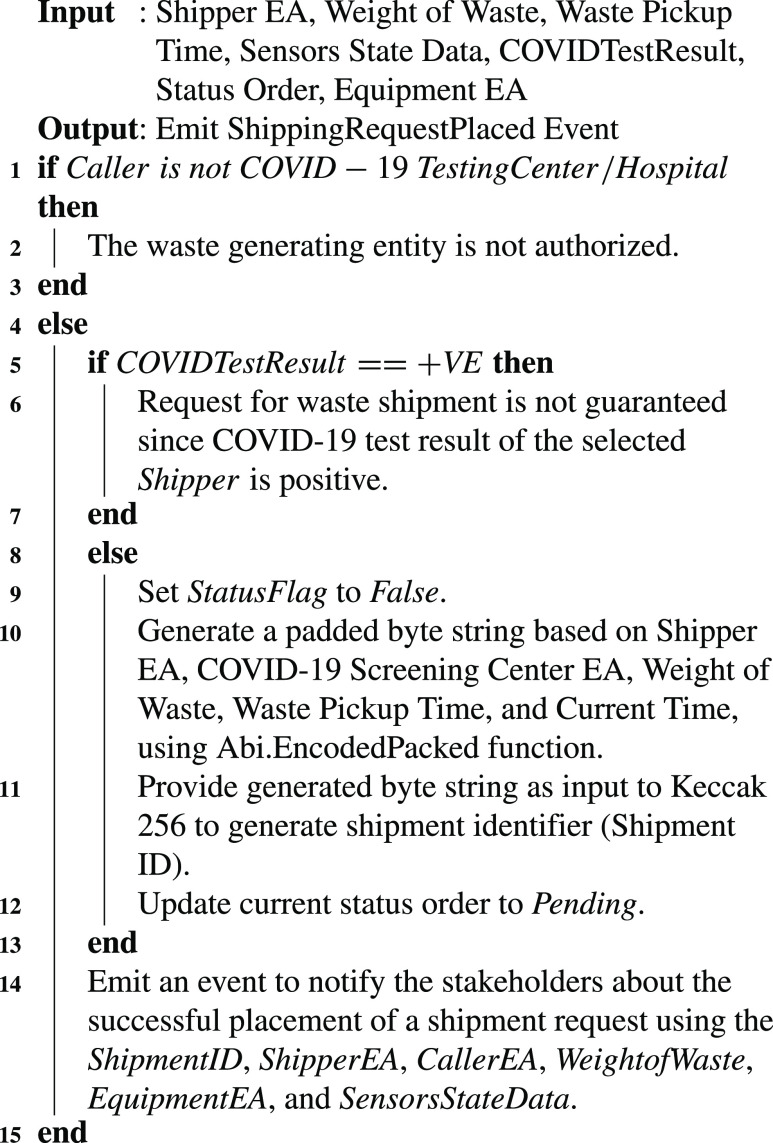
Initiating a Request for Shipment of the Medical Waste

The shipper confirms the shipment request of the COVID-19 screening center or hospital and starts the shipment. Algorithm 4 highlights the actions performed by the shipper during transportation of the waste related to COVID-19 medical supplies and equipment. Before data uploading, the system verifies that the current status of the waste shipment (line 1–2 in Algorithm 4) is accepted. After this verification process, it allows the shipper to periodically upload the shipment location and waste container’s state on to the chain until the waste reaches the medical waste treatment unit. It updates the shipment status to shipping to notify the related stakeholders about the shipment. On reaching the terminal of the waste treatment unit (identified using ShipmentReachedFlag variable), it updates the status of the shipment to “reached” to inform the participating entities. It stores the logs of the events about the location and status of the shipment.

**Algorithm 4:**
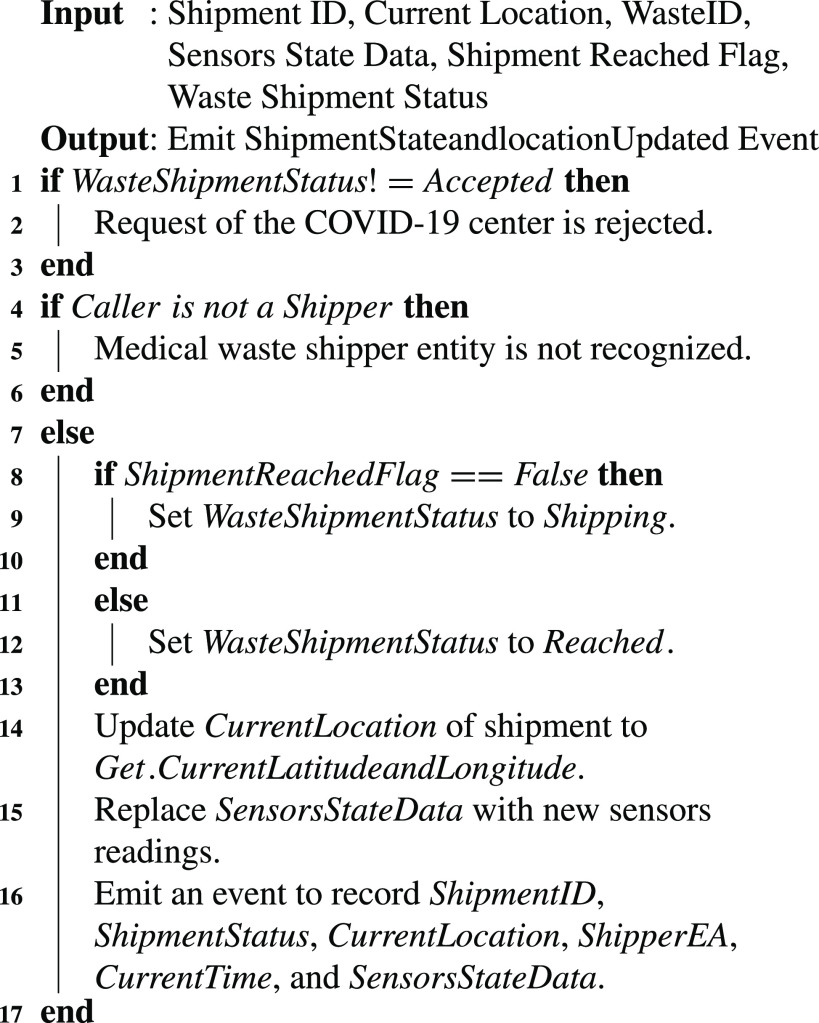
Monitoring and Storing Data Related to the Current Location and State of the Medical Waste

The rules to overcome COVID-19 from spreading have already been formulated to provide guidelines to the business personnel. Strict compliance with such rules by issuing penalties to the business stakeholders can significantly minimize the COVID-19 from spreading. Algorithm 4 successfully recorded the state of waste during its handling on the blockchain. Algorithm 5 analyzes the data related to the state of the waste during its handling and suggests penalties as well. It also proposes the FDA/Owner to invalidate the license of the relevant stakeholder. It uses the violation count and issues the penalties to the shipper if the violation count is greater than the threshold. Also, it verifies and issues a penalty to the user if the weight difference between shipped and received waste is different. After successful execution, it emits and event by triggering a transaction to record shipper EA, violations count, and shipper license ID to the blockchain.

**Algorithm 5:**
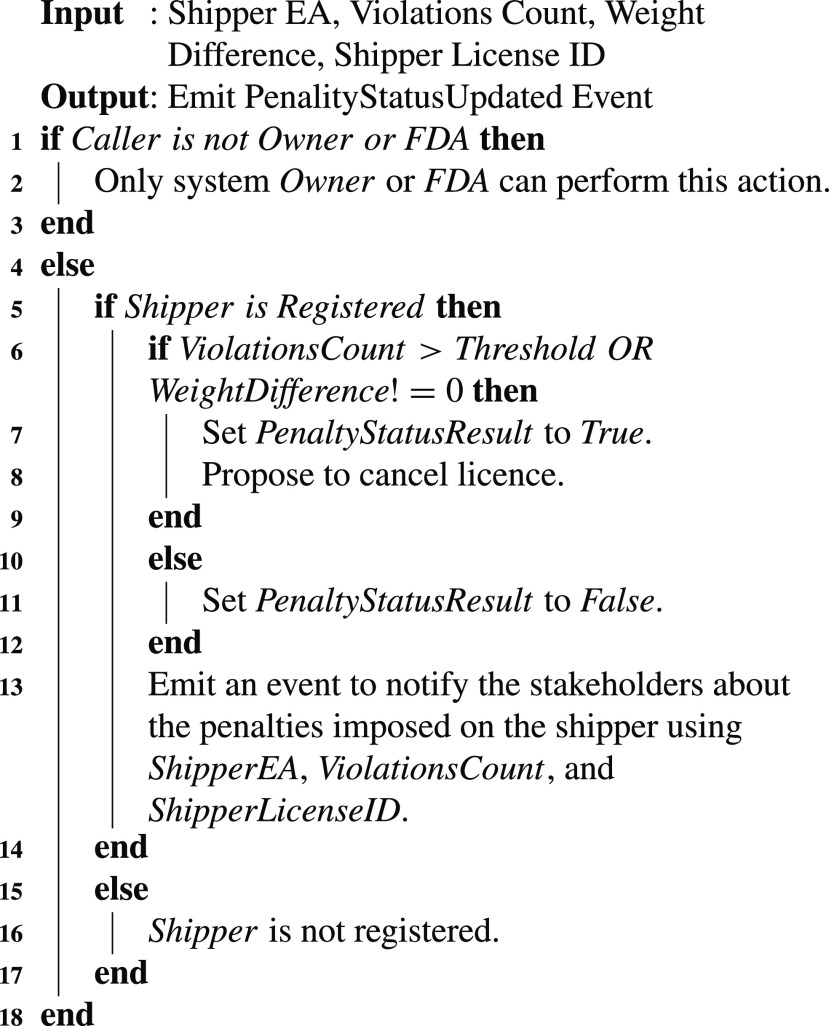
Auditing the Stakeholder Operations to Issue Penalties

## V. System Testing and Validation

In this section, we present system testing and validation details. We explain all the functions and their outputs when they are executed in the Remix IDE environment.

The proposed blockchain-based system is developed and rigorously tested to verify that the rules implemented in smart contracts are not violated during the business processes execution among system users. More specifically, it validates the behavior of functions implemented in smart contracts based on a set of inputs and scenarios. The modifiers are used for the function to assure that only authorized users can call a particular function. It generates an error when it recognizes unauthorized access to call a function. Many events are implemented in smart contracts that transparently record the logs of the transactions when they are executed. Finally, the entities of the system including smart contracts, system users, and medical equipment and supplies are uniquely identified using their Ethereum addresses. This system can be used to trace the medical equipment and supplies from their manufacturing till the waste disposing stage. The use of penalties for breaching the rules during COVID-19 waste handling can significantly control the spread of COVID-19.

Each entity in the system is represented by a unique Ethereum address. Table 1 highlights the Ethereum addresses of the participating stakeholders. Also, it has assigned Ethereum addresses to each medical equipment or supplies to identify it. For instance, we have assigned the “0x4B0897b0513fdC7C541B6d9D7E929C4e5364D2dB” Ethereum address to refer to the swab stick used to diagnose COVID-19. However, the system can be updated by employing barcodes or QR-codes as identifiers of the medical equipment and supplies to track them. The lot or batch of the medical equipment and supplies order is represented by a unique identifier generated by the Keccak256 algorithm.

**TABLE 1:** Ethereum Addresses for Participants

Stakeholder Name	Ethereum Address
Medical Equipment Manufacturer	0x78731D3Ca6b7E34aC0F824c42a7cC18A495cabaB
Distributor	0x4B20993Bc481177ec7E8f571ceCaE8A9e22C02db
COVID-19 Testing Center	0xAb8483F64d9C6d1EcF9b849Ae677dD3315835cb2
Medical Waste Shipper	0x617F2E2fD72FD9D5503197092aC168c91465E7f2
Medical Waste Treatment Facility	0x17F6AD8Ef982297579C203069C1DbfFE4348c372
FDA	0x5B38Da6a701c568545dCfcB03FcB875f56beddC4

### A. Forward Supply Chain of Medical Equipment and Supplies

This section presents a few results about the forward supply chain management of medical equipment and supplies to highlight the correct working of our proposed system.

**FIGURE 6.**
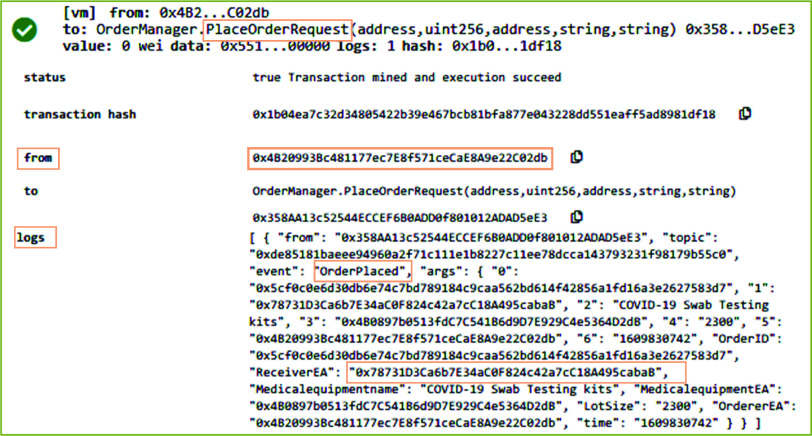
Logs showing the successful order placement by the distributor for medical equipment and supplies.

Fig. 6 shows the results on executing the *PlaceOrderRequest* function of the OrderManager smart contract in remix IDE. *PlaceOrderRequest* function enables the participants such as medical equipment distributor and COVID-19 testing center to register a request for system participants responsible to arrange and supply medical supplies and equipment such as COVID-19 swab testing kit. In the presented result in Fig. 6, *PlaceOrderRequest* function is triggered by the distributor stakeholder of the proposed system. The system validates the identity of the distributor to verify that it is a registered user and holds a valid license number. Since the distributor gets the medical supplies from the manufacturer, hence system verifies that the receiver of this function call should be the manufacturer stakeholder. On successful execution of this function, an event named Orderplaced is emitted that stores the transaction log on the blockchain. The transaction log includes medical equipment EA, OrderID, Equipment Name, requested LotSize, and address of the order receiver.

**FIGURE 7.**
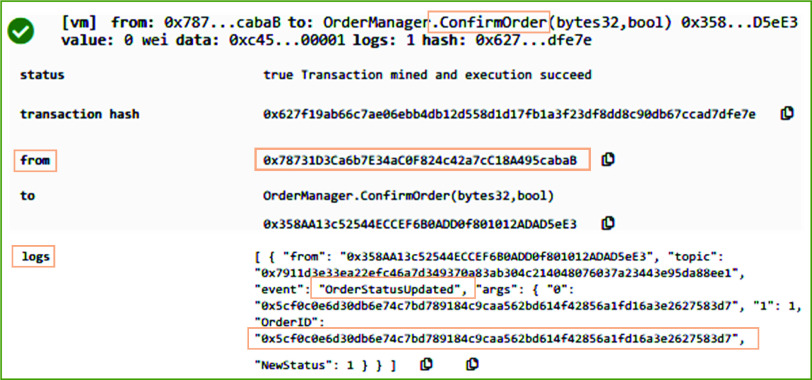
Logs showing the order confirmation by the manufacturer for medical equipment and supplies.

On successfully executing the *PlaceOrderRequest* function, the system sets the status of the transaction to pending, thereby enabling the users to view the current status of the order for medical equipment. The receiver of the order can examine the needs and requirements of the order request (in offline mode), and it notifies the orderer about its response using on-chain resources. Fig. 7 shows the result in executing the *ConfirmOrder* function of the OrderManager smart contract. The manufacture uses this function to publish its response by either accepting or rejecting the distributor request. This function is triggered by the manufacturer stakeholder only since the orderer entity in Fig. 6 is a distributor. On successful execution of the *ConfirmOrder* function, the manufacturer response is recorded on the blockchain by emitting an event called OrderReceived. This event stores the updated status of the order along with the OrderID that was generated by the *PlaceOrderRequest* function. The presented result in Fig. 7 shows that the medical supplies request transaction was successfully accepted by the manufacturer.

**FIGURE 8.**
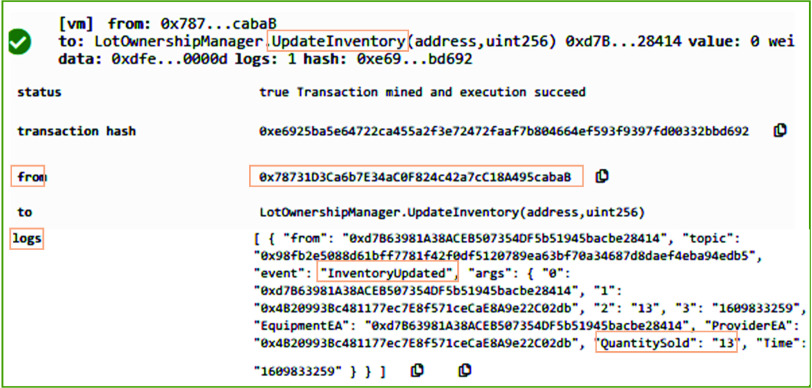
Logs showing the inventory updating about COVID-19 related medical equipment and supplies.

**FIGURE 9.**
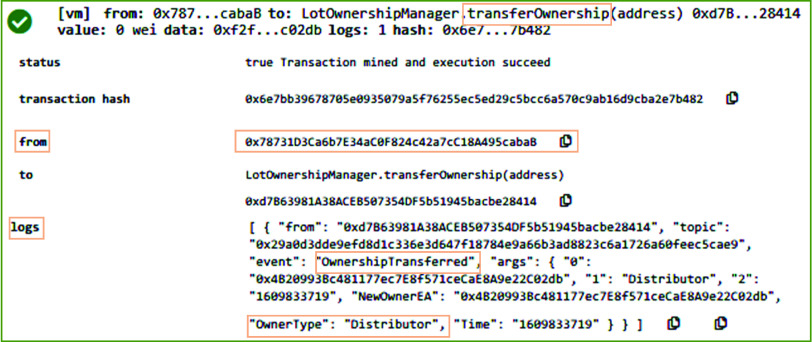
Logs showing the transferring of ownership to the distributor.

After accepting the order request, the manufacturer updates the record of medical supplies inventory based on the lot size requested by the distributor. In the orderManager smart contract, the *UpdateInventory* function is responsible to assure that the stock available in the store can fulfill the requirements of the distributor. Further, it updates the remaining medical supplies stock by subtracting the requested quantity from the available stock. On successful execution, as can be seen in Fig. 8, it emits *EquipmentSoldtoOrderer* event to record the logs on blockchain to show that medical supplies are sold out to the distributor. It records the address of the distributor, quantity sold, available quantity, Ethereum address of the medical supplies, and the time when this action is performed. After updating the inventory, the ownership is transferred and the distributor is updated as the new owner. Fig. 9 shows the result of executing the *TransferOwnership* function of the orderManager smart contract. The system verifies that only the shipper of the medical supplies can call the *TransferOwnership* function. In our case, this function was called by the manufacturer. On successful execution of this function, an event is emitted and it stored the type and Ethereum address of the new owner, e.g., distributor.

After successful execution of *TransferOwnership*, *ConfirmReceived* function is called by the medical equipment orderer (i.e., distributor). Before *ConfirmReceived* function execution, shipment order status must be previously set as accepted. On successful execution of this function, an event called OrderReceived is emitted that logs the OrderID.

**FIGURE 10.**
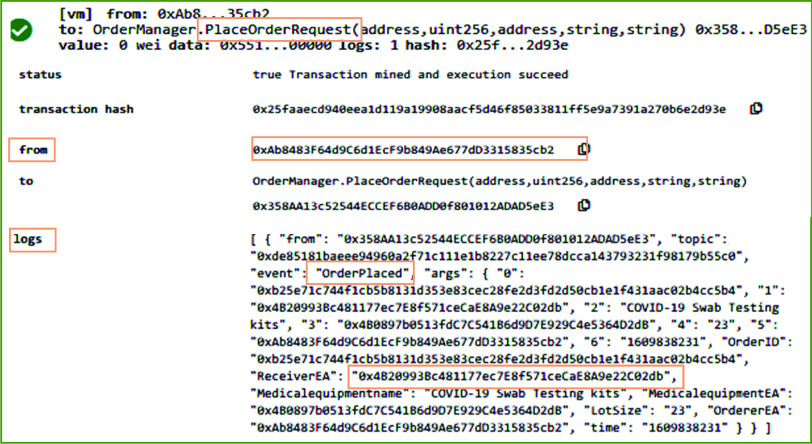
Logs showing medical equipment and supplies order placement by the COVID-19 testing center.

Fig. 10 refers to a scenario wherein the order for medical equipment and supplies is placed by the COVID-19 testing center. Fig. 10 shows the result of executing the *PlaceOrderRequest* function in RemixIDE. In contrast to Fig. 6, the originator of the transaction in Fig. 10 (See “from” part) is the COVID-19 testing center. After successful execution of this function, an event is generated that records the transaction log by immutably storing the Ethereum address of the orderer, medical equipment, and receiver, Lot size, and orderID to uniquely identify the order. After successful execution of *PlaceOrderRequest* function, the medical supplies distributor calls *ConfirmOrder* function to confirm the medical equipment supplies order.

**FIGURE 11.**
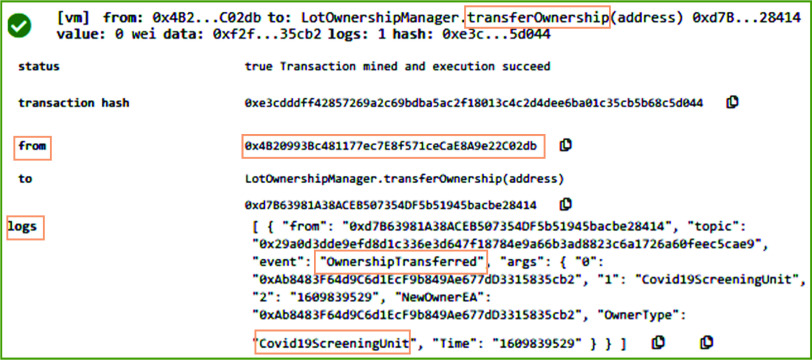
Logs showing the ownership transferring to the COVID-19 testing center.

After successful order confirmation, the *UpdateInventory* function of the Ordermanager smart contract is called. On successful execution, the log of the transaction is generated and it transparently stores COVID-19 testing center as the receiver of the order in addition to many other important parameters such as quantity sold and remaining quantity, and the orderID. On successful execution of *TransferOwnerhip* function, the Ethereum address of the new owner of medical supplies is immutably recorded on the blockchain as can be seen in Fig. 11.

### B. Waste Management of Medical Equipment and Supplies

Many medical equipment and supplies such as personal protective equipment and swab testing tubes, are specially designed to be used for one time only. Thus, these equipment require immediate disposal after their usage. Considering the contagious nature of coronavirus, the COVID-19 related medical waste demands to be carefully handled by the stakeholders. Our proposed system has implemented a shipment handling smart contract to assure that the medical waste shipper should have a valid COVID-19 result to ship the medical waste. The system is programmed such that it triggers an error on selecting a medical waste shipper who does not have a valid COVID-19 result. The error occurs in response to the COVID-19 testing center manager’s interaction with the *PlaceShipmentRequest* function.

**FIGURE 12.**
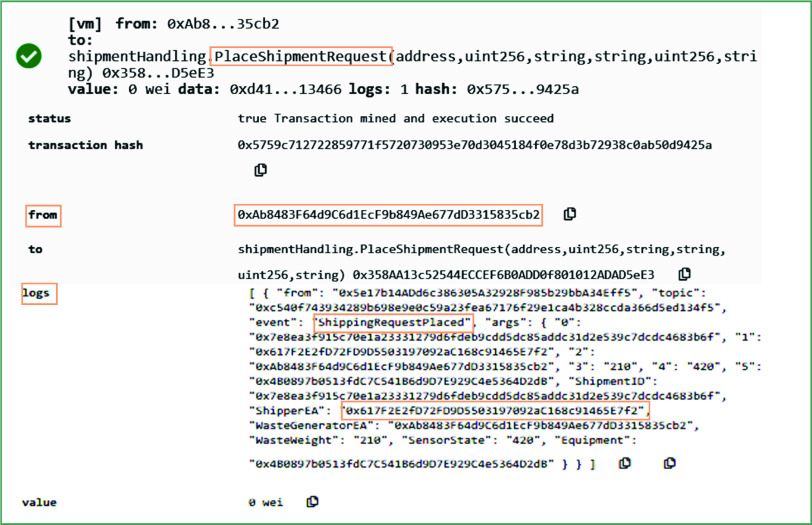
Logs showing the placement of request to ship COVID-19 medical waste.

Fig. 12 shows the result on calling a *PlaceShipmentRequest* function by the COVID-19 testing center. This function accepts the shipper’s Ethereum address along with the weight of COVID-19’s waste, earliest pick-up time, sensors data attached to the waste bags, and the hashes of the waste certificates and 3D images. It proceeds further only if the shipper has a valid COVID-19 result. It sets the transaction status to pending to notify the shipper that the shipment services for medical waste are required (for traceability purposes). On successfully executing this function, it emitted an event and records the Ethereum address of the shipper and waste generator, shipment ID, and other necessary details on the blockchain. The shipment request is examined by the shipper to either accept or reject it based on the function’s input parameters. After accepting the medical waste shipment request, the shipper collects the waste and routes it to the medical waste treatment facility.

**FIGURE 13.**
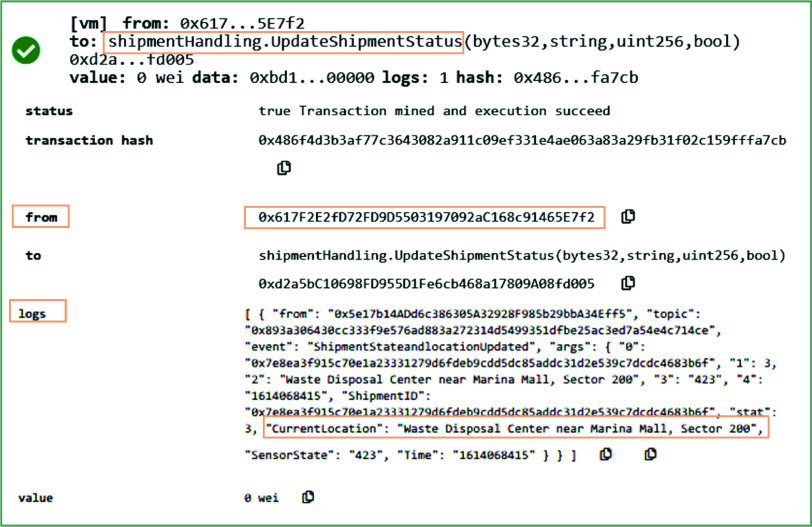
Logs showing the updating of location and state of the medical waste during its shipment.

Fig. 13 shows the result on calling a *UpdateShipmentStatus* function. This function can be called only if the medical waste shipper has already accepted the waste shipment request. It continuously records the updated data (company’s local server) related to the current location, state, and status of the shipment. This data is periodically stored on the blockchain. It emits the event called shipmentstatusandlocationupdated to record the current location and state of sensors to notify the users about the current location and state of the shipment. The state of the shipment enables the users to know that at which time and place the sealed bags of COVID-19 waste were unsealed.

**FIGURE 14.**
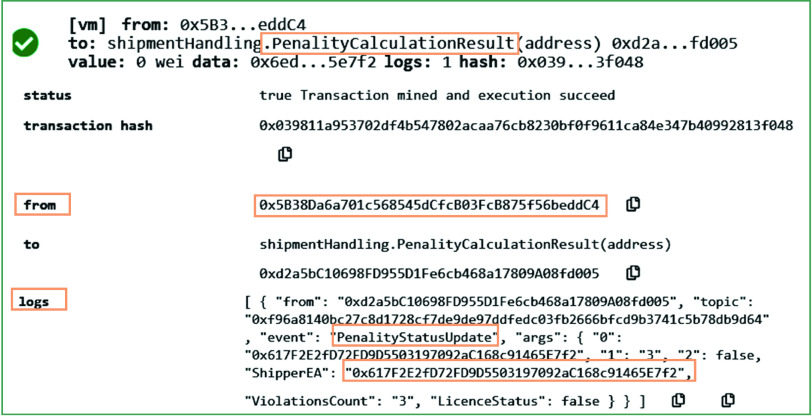
Logs showing the successful and rules-based shipment of medical waste.

The COVID-19 related waste is usually packed in multiple boxes that are carefully sealed to minimize the chances of spreading COVID-19 during its shipment stage. Many sensors are mounted inside such boxes to identify any human attempt made to open and stealing them (if any). The deployed sensors can sense, monitor, and record this data on to the blockchain ledger. Fig. 14 shows the results on calling the *PenaltyCalculationResult* function. This function can be called by the owner of the smart contracts. As can be seen in Fig. 14, no penalty is issued to the medical waste shipper since no non-compliance with rules for COVID-19 waste handling is identified. On successful execution of this function, an event has been emitted that can store whether the license of the shipper is canceled or not based on the violations count.

**FIGURE 15.**
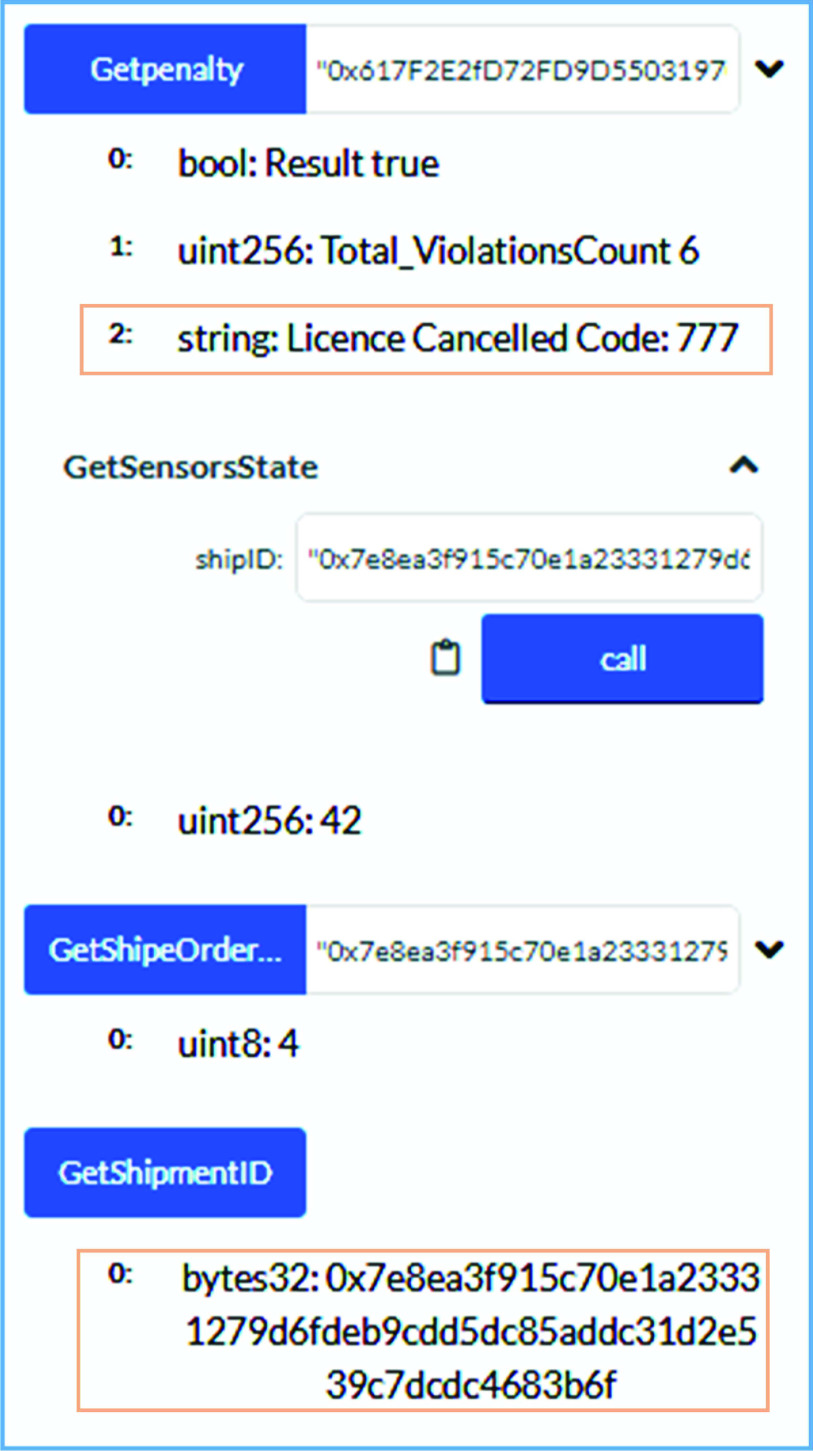
Highlighting the total violations count and license cancellation of shipper on calling *GetPenaltyStatus* function.

In contrast to Fig. 14, Fig. 15 presents the results in which a penalty in terms of cancellation of license of a stakeholder has been issued to the COVID-19 medical waste shipper. Once the shipper’s license is canceled, he/she cannot further participate in the shipping services. Fig. 15 shows graphically the results on executing the *PenaltyCalculationResult* function. It also shows that four violations are reported during the medical waste shipment. It is to note that we have set the threshold value to three to trigger the license cancellation event for the shipper.

## VI. Discussion

This section presents the cost and security analysis of the proposed solution. It also outlines the future research challenges and generalization.

### A. Cost Analysis

The Ethereum blockchain platform meters the transaction computations in the unit of gas. The user is required to set a maximum amount of gas for each transaction to execute it. Each Ethereum virtual machine (EVM) operation consumes a certain amount of gas. Also, in smart contracts, storage variables, loops, nested decision making, mappings, and manipulations share the major portion of gas consumption. During transaction execution, if the required amount of gas is higher than the set Gas-limits then EVM halts the operations and charges the users to make sure that non-terminating computations cannot be performed to slow down the network. Because of the high flexibility, Ethereum allows the users to specify the cost of gas consumption based on their requirements (slow or fast execution). Hence, users can offer more gas price for the miners to prioritize their transactions. The Remix IDE calculates and stores the transaction and computation cost as part of the transaction log [27], [28]. The gas prices can be easily specified in Gwei. Gwei is the most known unit of ether. An analysis of a smart contract or method for gas consumption can be helpful to design lightweight and computationally inexpensive smart contracts.

Ethereum Gas Station estimates the gas price for the Ethereum-based transactions. It offers different speeds of executing the transactions that are largely affected by the gas price offered by the user [27], [29]. For example, it can offer a price for the given units of gas based on fastest, fast, average, and slow transactions execution speed. The gas price in Ethereum is variable since it changes over time and highly depends on the congestion in the network. Hence, the gas price could be different during different times of the day. The current research has used the gas prices offered by the ETH Gas station on 26 December 2020. At the time of writing of this paper, the transaction fee for 21000 gas units is observed 58, 45, and 38 Gwei for fastest, average, and slow transaction execution speeds. Moreover, to convert Ethers into USD, we used the conversion rate from May 2020 to represent a more stable and realistic value.

Table 2 shows both transaction and computations gas units consumed by functions of the proposed smart contracts when executed in Remix IDE. It also shows the transaction fee for the fastest, average, and slow transaction execution speeds for each function. The fastest transaction execution speed requires more Ethers than slow or average execution mode. Among all the specified functions in Table 2, *PlaceShipmentRequest* and *PlaceOrderRequest* need the highest number of tokens for their successful execution since these functions have involved various complex operations. For instance, *placeShipmentRequest* function has implemented several structures, mappings, manipulations, and modifiers to assure the correct business operations among participating stakeholders. It also has implemented many modifiers to assure that, (1) the potential medical waste shipper has no COVID-19 symptoms, (2) the caller of the function is a business representative of COVID-19 testing hospital, and (3) the shipper has a registered license number. It also implements structures to record the details about the medical waste material and shipper. The *GetOrderID* function requires the least number of Ethers for its execution among all functions.

**TABLE 2:** Gas cost in Ether of the functions in smart contracts.

Function Name	Transaction Gas	Execution Gas	Fastest Execution (Ether)	Avg. Execution (Ether)	Slow Execution (Ether)	Transaction Cost (USD)
RegisterTestingCenter	65893	43021	0.0038218	0.0029652	0.0025039	0.441763077
RegisterDistributor	65927	43055	0.0038238	0.0029667	0.0025052	0.441992436
WasteShiperExists	23900	1220	0.0013862	0.0010755	0.0009082	0.160233726
DistributorlicenseisValid	24878	2198	0.0014429	0.0011195	0.0009454	0.166796922
PlaceOrderRequest	222507	196691	0.0189131	0.015353	0.0106803	1.884325329
GetOrderID	22293	1021	0.001293	0.0010032	0.0008471	0.149453853
ConfirmOrder	71979	48339	0.0041748	0.0032391	0.0027352	0.482571336
GetOrderState	24693	1245	0.0014322	0.0011112	0.0009383	0.165544269
ConfirmReceived	36082	12634	0.0020928	0.0016237	0.0013711	0.241903173
UpdateInventory	43466	20594	0.002521	0.001956	0.0016517	0.291409431
TransferOwnership	62136	39456	0.0036039	0.0027961	0.0023612	0.416586516
UpdateHealthStatus	51285	28477	0.0029745	0.0023078	0.0019488	0.343826784
PlaceShipmentRequest	181512	156464	0.0105277	0.008168	0.0068975	1.216925925
GetShipmentID	22339	1067	0.0012957	0.0010053	0.0008489	0.149771427
ConfirmShipmentRequest	62151	38511	0.0036048	0.0027968	0.0023617	0.416674731
ConfirmShipmentReceiving	40384	16936	0.0023423	0.0018173	0.0015346	0.270749478
GetPenalty	31353	8673	0.0018185	0.0014109	0.0011914	0.210198702
UpdateShipmentStatus	27881	3409	0.0016171	0.0012546	0.0010595	0.186927585
GetWasteState	24583	1135	0.0014258	0.0011062	0.0009342	0.164820906
PenaltyCalculationResult	41538	18858	0.0024092	0.0018692	0.0015784	0.278477112

### B. Security Analysis

Herein, we present a detailed security analysis of the proposed solution that offers high security, robustness, and resilience features. Below is given a brief discussion on the key security and privacy issues that are addressed in our system. It also discusses the security analysis of the implemented smart contracts.

#### 1) Availability and Fault Tolerance

Blockchain technology has gained tremendous popularity in the healthcare sector because of its inherited security features. Unlike centralized-based systems, the decentralized feature makes it highly acceptable and dependable to healthcare organizations. Furthermore, the decentralization makes the system highly fault-tolerant and available despite the occurrence of malicious codes such as the denial of service (DoS) attacks. Therefore, such high availability makes it acceptable to the industries. Since our system has been implemented on the Ethereum blockchain platform, the services of our system will always remain available to the participating stakeholders.

#### 2) Data Integrity and Immutability

Data manipulation is one of the serious threats to the integrity of the data. Among many, approaches such as append-only, data certification, and sophisticated access control can enable organizations to protect their data. In the blockchain, through cryptographic functions, data integrity is preserved. The hashing algorithms make the blockchain transactions immutable. In our proposed waste management system, the transactions of the participants such as the manufacturer and COVID-19 testing center are digitally signed before they are written on the blockchain. Thus, the immutability feature of blockchain assures that these transactions cannot be edited, modified, or deleted. Hence, the proposed solution preserves data integrity.

#### 3) Non-Repudiation and Digital Signatures

Digital signatures are based on mathematical schemes and are often used to verify the authenticity of a transaction. Also, digital signatures provide non-repudiation service to the existing systems, since the signer cannot deny that he/she did not sign the transaction. In the Ethereum blockchain platform, each transaction of the user is secured by the digital signature. For instance, a waste treatment facility might have sent a waste shipment confirmation transaction to the hospital. The blockchain assure that the transaction is signed with the private key of the waste treatment facility. This transaction cannot be modified or deleted because of immutable blockchain technology. At any time, this transaction cannot be denied by the waste treatment facility since the originator of the transaction can be verified by the signature.

#### 4) Resilience Against Man-In-Middle Attacks

In a Man-in-middle attack, a user intercepts communication between two parties to secretly eavesdrop or modify the exchanging data. Blockchain technology assures that no man-in-middle attack can be successfully launched. It requires the users to sign digitally the encrypted transactions to immutably store it on the blockchain platform. The launch of the Man-in-middle attack is successful only if the malicious actor has successfully retrieved the private key of the communicating parties. The miners of the Ethereum blockchain platform cannot approve those transactions wherein signatures do not match with the signatures of the legitimate users. Our system is comprised of several stakeholders who are often located in geographically different locations. Thus there is a high probability of Man-in-middle attacks. Since our system is implemented on the Ethereum blockchain platform, hence it is safe against Man-in-middle attacks.

#### 5) Protection Against Smart Contract Vulnerabilities

An ill-designed or poorly written smart contract can exhibit low performance, limited security, and high transaction execution fee. It is highly recommended to write smart contracts carefully to avoid any exploitation of smart contracts for malicious activities. There are many tools available that statically analyze the smart contracts (e.g., SmartCheck and Securify) and provide guidelines to improve the cost, performance, and security of smart contracts. The guidelines state that smart contracts should be checked for infinite loops, floating-point operations, reentrancy errors, and common bugs that can make the smart contract highly vulnerable to attacks. We improved our smart contracts by analyzing them using the SmartCheck [30], [31] tool. Our smart contracts have shown no bugs and vulnerabilities such as infinite loops, race condition, integer division, Denial of Service (DoS) by an external, malicious external library, and locked transfers. Table 3 enlists the potential vulnerabilities in code and attacks that can exploit them to greatly deteriorate the performance of the smart contracts.

**TABLE 3:** Attacks and Code Vulnerabilities That Affects the Performance of Smart Contract Applications

Attacks/Vulnerabilities	Description
REENTRANCY	Reentrancy attacks are mainly of two types i.e., single and cross-function reentrancy attacks. A single function reentrancy attack involves a recursive call to the function itself. Alternatively, a cross-function attack occurs on calling a function of another contract through an outer call to drain any available balance from the contract.
OVERFLOW AND UNDERFLOW	By exploiting this attack hackers can be successful to own more funds than what they have in their wallets. It is a safe programming practice to include integer overflow and underflow checks to the variable to assure that such variables remain in their range after the execution.
SHORT ADDRESS ATTACK	This attack mainly targets EVM, and it is similar to the structured query language injection attacks in its nature. This vulnerability usually occurs from the sender's side because of the weak transaction generation code. On receiving a short address from the sender, Solidity appends zeros to the address to complete it; however, Solidity contracts treat them as data.
DEFAULT VISIBILITIES	The visibilities of functions represented by appropriate modifiers in smart contracts define who has the right to call the function. The default visibility of the functions in Ethereum smart contracts is public. It is a safe practice to protect the function from unauthorized access by explicitly declaring the visibility of functions.
TIMESTAMP DEPENDENCE	This vulnerability is usually exploited by corrupt miners to gain financial benefits. The corrupt miners can re-arrange the timestamp by a few seconds to get the benefits. It causes issues when the smart contract depends on the block timestamp to perform certain operations (e.g., sending Ethers).
BLOCK GAS LIMIT	Setting the limit to a block gas can guarantee that block will not grow infinitely, thereby limiting the transaction execution speed. Hence, the transactions with large gas consumption rates do not fit in a block and wait for an indefinite time. Applying loops on variable size arrays that grow with time is one example of such vulnerabilities that can assist the hackers to make a smart contract run out of the gas for their purposes.
THE DAO ATTACK	The DAO attack is an example of a successful attack that helped attackers in stealing Ethers from the wallet of the DAO. The attacker has exploited several bugs in the smart contract, such as reentrancy on a single function, reentrancy on cross- functions, exception handling, unsecured balance, and reentrancy on external contract functions. More specifically, the splitDAO’ function of the smart contract had recursive send pattern vulnerabilities that helped the attacker to steal Ethers from the DAO wallet.
MULTI-STAGE ATTACK	The default modifier of the smart contract functions is public. The multi-stage attack works strongly on encountering no access modifier to the functions of the smart contract. Also, using an internal modifier with functions can handle this attack in the Ethereum platform.

### C. Future Research Challenges

In this subsection, we enumerate and discuss several important challenges remaining to be addressed.

#### 1) GDPR and Data Privacy

Blockchain accomplishes data security through transparency and storing data on multiple nodes. However, the transparency and distributed storage of data can greatly affect data privacy. The public blockchain platforms face data privacy assurance challenges since all transactions and data are publicly available on the ledger, thus affecting the privacy of user data. The EU General Data Protection Regulation (GDPR) laws protect the data related to the organizations that participate in business processes in the forward supply chain and waste management of medical equipment and supplies. Our study has used a registration smart contract and function modifiers to control data access on the chain. Our proposed smart contracts can be modified and implemented on private permissioned blockchain platforms such as Hyperledger Fabric, Hyperledger Besu, and Quorum, [32] thereby preserving the privacy and confidentiality of waste processing and recycling data. Hyperledger Fabric ensures confidentiality and privacy of data through private channels that represent a private blockchain overlay. Nevertheless, in Hyperledger Besu, data confidentiality and privacy is guaranteed through a private transaction manager called Orion node. Quorum uses a private transaction manager called Tessera to implement private transactions to ensure data privacy [33].

#### 2) Regulation

Blockchain technology offers many advantages to the stakeholders involved in the forward supply chain of medical equipment and their disposal in terms of lower transaction cost, streamlined operations, improved system efficiency, data traceability, audit, and operational transparency. Blockchain is an emerging technology that lacks in providing certain specifications and surety required to the business organizations to adopt the new technology. For instance, integrating blockchain-based systems with legacy systems is extremely challenging. Also, the lack of regulation, awareness, understanding, governance, and developers’ support are some of the imperative challenges that should be addressed in the future.

#### 3) Smart Contracts Vulnerabilities and Attacks

Bugs and vulnerabilities can affect a smart contract’s normal functioning and behavior. Vulnerabilities in the code of smart contracts are often exploited by hackers to perform harmful activities. The examples of vulnerabilities in the code of the smart contract include reentrancy, overflow and underflow, unsecured balance, unchecked and failed send, non-validated arguments, short address attack, default visibilities, timestamp dependence, call stack depth limitation, multi-stage attack, prodigal contracts, transaction ordering dependency, and block gas limit [31]. Our developed smart contracts are safe against recursive reentrancy attacks as our implementation has not used any recursive call. Also, integer overflow and underflow checks are used with critical variables to avoid the vulnerabilities caused by integer overflow and underflow issues. The vulnerabilities caused by the default visibilities cannot occur since we have explicitly defined the access modifier with the functions. Finally, the current research work has not used the short addresses to avoid the issues caused by them. This research is mainly focused on analyzing the feasibility of the applicability of blockchain technology in digitizing the services related to the forward supply chain and waste management for COVID-19 medical equipment and supplies. Thus, it has not focused on making smart contracts unbreakable against all types of attacks.

#### 4) Throughput and Latency

Blockchain nodes use adequate time and energy to arrange and validate the transactions, store them, and create and broadcast each block to other mining nodes for verification and updating. The throughput of the Ethereum 1.0 platform is 16 to 20 transactions per second. Nevertheless, Ethereum 2.0 promises a throughput of 100,000 transactions per second [35]. The transaction latency in the Ethereum platform highly depends on the transaction fees and total convergence time of the implemented consensus algorithm including Proof of stake (PoS) and Proof of work (PoW). Regarding the nature of the forward supply chain and waste management processes implemented in our system, the throughput of the existing Ethereum platform is enough to successfully execute such processes because the system stakeholders do not require real-time transaction processing. Hence, the stakeholders of our proposed system do not generate blockchain transactions at high speed. Since our proposed system is implemented on the Ethereum blockchain platform, the throughput and latency of the developed system are the same as the throughput and latency of the existing Ethereum blockchain platform.

#### 5) Interoperability

The stakeholders involved in the forward supply chain and waste management of medical equipment and supplies could be practicing multiple platforms to do their business activities. The interoperability feature in existing blockchain-based solutions provides cross-platform communication ability to increase system productivity and throughput [36], [37]. The difference in consensus protocols, implemented security schemes, registration services, governance model, and supported languages are the primary challenges in developing an interoperable platform. It is desired that the involved stakeholders in forward supply chain and waste management activities should be using an interoperable blockchain platform. Our system is generic that can be customized for any platform with little effort since its code is publicly made available.

#### 6) Scalability

The notable increase in the transaction rate of blockchain platforms can influence the average response time and transaction fees. Consequently, the increased transaction rate can make the blockchain remarkably slow and unstable [38]. The complex, highly fragmented, and dynamic supply chain and waste management systems can generate an ample amount of data, thereby causing a delay in transactions execution time and throughput. In our proposed system, Algorithm 4 is the most expensive part as it has involved numerous state parameters and operations to periodically calculate and store the data on the blockchain. Highly scalable platforms that offer strong security features and throughput can be used for system deployment to enrich the user’s experience.

#### 7) On-Chain and Off-Chain Governance

Blockchain is governed and run by several communities including, core developers, node operators, token holders, and blockchain teams. Being an evolving technology, mature strategies that can efficiently govern blockchain are lacking. Broadly, governance models are classified into two types such as on-chain and off-chain blockchain governance. The Ethereum platform follows an off-chain governance model. The on-chain governance model can leverage the voting mechanism to achieve direct democracy in blockchain affairs. Tezos and hard fork are on-chain and off-chain governance strategies, respectively [39]. The consistently changing blockchain platforms, due to governance strategies, could affect the security and performance of the developed systems.

### D. Comparison With the Existing Forward Supply Chain and Waste Management Solutions

Table 4 presents a comparison between conventional froward supply chain and waste management solutions and our approach based on several design parameters and opted technological aspects. It shows the advantages of our solution compared to the existing solutions owing to the integration of IPFS with Ethereum blockchain platform to manage large-sized waste management data, enforcement of trust among participating stakeholders using fast, secure, and self-executing smart contracts, and transparency in supply chain operations (forward) to assure that the COVID-19 medical equipment and supplies are genuine and not fabricated in any way. The proposed solution has achieved all the set requirements such as traceability of COVID-19 medical equipment and supplies and their waste, compliance with rules designed for COVID-19 medical equipment and supplies waste shipment to minimize COVID-19 from spreading, supply chain transparency, and frauds identification related to the illegal waste shipment. As highlighted in Table 4, existing works ([10], [17], [18], [34]) have mainly focused on the management of waste bins responsible to monitor, collect, and transfer data related to domestic and electronic waste. Also, the researchers in [22] have focused on the activities involved in the procurement of PPE.

**TABLE 4:** Comparison of Our Approach With the Existing Solutions

Study	Waste Type	Shipping Rules	Traceability	Decentralized Storage	Waste Frauds	Supply Chain
[17]	Domestic waste	No	Yes	No	No	No
[18]	Electronic waste	No	Yes	No	No	Forward
[10]	Domestic Waste	No	No	No	No	No
[22]	N/A	No	Yes	Yes	No	Forward
[34]	Domestic Waste	No	No	No	No	No
Our Study	COVID-19 medical equipment waste	Yes	Yes	Yes	Yes	Forward

### E. Generalization

We present a working prototype of a blockchain-based system along with its security and cost analysis to investigate the feasibility of employing blockchain technology in the forward supply chain and waste management of COVID-19 related medical equipment and supplies. The proposed system is designed and tested on a public Ethereum blockchain platform to meet the high visibility, transparency, and security requirements of users of supply chain and waste management industries. Also, such users do not have fast and real-time transaction processing requirements. Since data and transactions stored on public blockchain platforms are encrypted, thereby enforcing trust which makes it more appealing to business organizations to store their transactions. The proposed system can be customized to meet the growing demands and requirements of various organizations. To provide high transaction execution speed, privacy, transparency, and security, one can modify the proposed smart contracts with few efforts to deploy them on private blockchain platforms. Even, our system can be integrated with external oracles and proxy encryption schemes to further improve the privacy-related issues.

The proposed system can efficiently trace and track the forward supply chain of medical equipment supplies related to COVID-19. It also assists in tracing and tracking the medical waste caused by medical equipment and supplies and assures that the medical shipped waste complies with COVID-19 safety rules. It employs sensors to monitor and ensure that sealed medical waste bags cannot be exposed during shipment to minimize the COVID-19 from spreading. It cancels the shipper license for violating the rules specifically designed to decrease the chances of COVID-19 from spreading during waste transportation. It also assures that medical waste found missing is immediately reported by the medical waste treatment facility.

COVID-19 inherits many similar characteristics of many other viruses or diseases that belong to a similar category (e.g., contagious and human-to-human transmittable). Since the proposed solution has successfully traced and tracked the forward supply chain and waste related to medical equipment and supplies; therefore, this research is equally valid to manage the forward supply chain of medical equipment for other diseases too. For example, the authorities can use this research to trace and track the medical supplies for Influenza (Flu). In addition to the forward supply chain and waste management of medical equipment and devices, this research is valid to trace and track any vaccine for COVID-19 treatment. The examples of other beneficiaries of this work include drug dealers, pharmacists, telehealth specialists, telemedicine, regularities, health authorities, and health professionals.

The smart contracts are implemented in solidity for the Ethereum-blockchain platform. Since our solution is generic; therefore, one can easily transform it with minimal efforts for other blockchain platforms. As our solution can successfully minimize the spread of COVID-19 during the forward supply chain and waste handling, hence healthcare authorities can extend it by introducing new laws to further minimize the spreading of COVID-19.

## VII. Conclusion

In this paper, we have designed, developed, and evaluated a blockchain-based solution to automate forward supply chain processes and establish data provenance about the COVID-19 medical equipment and their waste disposal. We developed four smart contracts and proposed five algorithms to implement all functionalities and trigger events and notifications. We integrated the Ethereum blockchain with decentralized storage of IPFS to provide a secure, transparent, auditable, reliable, traceable, and trustworthy solution for the forward supply chain and waste management of COVID-19 medical equipment. The proposed approach can assist authorities in assuring that the COVID-19 medical waste is disposed of properly, and COVID-19 testing centers are using genuine medical equipment to treat COVID-19 patients. We presented a detailed cost analysis to show the affordability of the proposed approach. We also evaluated the proposed approach against known vulnerabilities using the SmartCheck software. The proposed approach is generic and applicable to various use case scenarios with minimal modifications. Our implementation code is made publicly available on GitHub. In the future, we aim to design and implement an incentive system for the COVID-19 treatment hospitals to dispose of medical waste.
